# Residual Efficacy of Two Diatomaceous Earths from Greece for the Control of *Sitophilus oryzae* (L.) (Coleoptera: Curculionidae) and *Rhyzopertha dominica* (F.) (Coleoptera: Bostrychidae) on Wheat and Maize

**DOI:** 10.3390/insects15050319

**Published:** 2024-04-30

**Authors:** Georgia V. Baliota, Christos I. Rumbos, Christos G. Athanassiou

**Affiliations:** Laboratory of Entomology and Agricultural Zoology, Department of Agriculture, Plant Production and Rural Environment, University of Thessaly, Phytokou Str., 38446 Nea Ionia, Magnesia, Greece; crumbos@uth.gr (C.I.R.); athanassiou@agr.uth.gr (C.G.A.)

**Keywords:** inert dusts, natural insecticides, stored-product insects, granulometry

## Abstract

**Simple Summary:**

Natural insecticides are currently given high priority in Integrated Pest Management (IPM) protocols. To this end, diatomaceous earth (DE) has gained a lot of attention as an environmentally friendly alternative to conventional insecticides that can successfully repel and control a diverse variety of the most important stored-product insect pests, such as the species *Sitophilus*, *Rhyzopertha*, *Tribolium*, and others. However, most published research about the DEs’ insecticidal efficacy has been conducted in simplistic short-term laboratory experiments, excluding the evaluation of the persistence of such ingredients in grain applications. In this study, we evaluated two different DE formulations derived from a single deposit from Greece, when applied in wheat and maize against two primary stored-product insect pests, *Sitophilus oryzae* (L.) (Coleoptera: Curculionidae) and *Rhyzopertha dominica* (F.) (Coleoptera: Bostrychidae). Based on the results, the DE application was effective and persistent as a grain protectant against major stored-product insects for a storage period of six months. This study provides more evidence for the successful utilization of these natural insecticides for long-term protection of stored products from insect infestations, as an alternative to residual insecticides and fumigants that are commonly used in stored-grain protection systems or as part of integrated pest management (IPM) strategies.

**Abstract:**

We evaluated the persistence and efficacy of two different, in granulometry and content of diatoms, diatomaceous earth (DE) formulations (i.e., DE5 and DE6), against two major beetle species of stored products, i.e., *Sitophilus oryzae* (L.) (Coleoptera: Curculionidae) and *Rhyzopertha dominica* (F.) (Coleoptera: Bostrychidae). The formulations were applied as powders in soft wheat and maize in two doses of 500 and 1000 mg kg^−1^ (ppm). Samples of the treated grains were taken on the day of application and every 30 days until completion of the six-month period of storage. Adults of *S. oryzae* and *R. dominica* were exposed to the treated grains at 25 °C and 55% relative humidity, and the mortality was measured after 7, 14, and 21 days of exposure. *Rhyzopertha dominica* survival was not affected by any combination of DE formulation, dose, and commodity. Contrariwise, the DEs caused significant adult mortality of *S. oryzae*, in most of the cases tested. We observed that DE6 was equally effective in both wheat and maize, and no considerable variations were observed in *S. oryzae* mortality during the 6-month experimental period. Furthermore, DE6 was more effective against *S. oryzae* than DE5, a difference that could have potentially contributed to the variations in the diatom granulometry between these two DEs. Thus, a DE treatment of 1000 ppm was shown to provide long-term protection of wheat and maize against *S. oryzae*, but this is strongly dependent on the DE formulation, commodity, and insect species. Overall, such natural resource-based inert silicaceous deposits could be used with success in stored-product protection with only some minor modifications, such as sieving and drying of the raw deposit.

## 1. Introduction

At present, the majority of pest management practices rely on residual synthetic insecticides. However, it is essential to enhance these approaches by including more sustainable and environmentally friendly treatments for both raw and processed commodities [1]. To this end, diatomaceous earths (DEs) have been long considered viable alternatives to conventional insecticides in stored-product protection, given their low toxicity to mammals, beneficial organisms, and the environment [1]. Moreover, the use of DEs has been evaluated in numerous studies, especially during the last two decades, under different application scenarios in laboratory, semi-field, and field experiments, with good results for a wide range of insect species [2,3,4]. Currently, a large number of DE-based formulations have been registered for the control of insects that infest stored products, while the registration of DEs for this use is currently much easier than that of conventional grain protectants [1]. Moreover, DEs can provide a satisfactory level of grain protection in geographical zones where access to conventional insecticides is not easy, such as sub-Saharan Africa [4,5].

One of the most important advantages of the use of DEs in stored grains is their negligible mammalian toxicity, which, along with their natural origin, makes these agents ideal for organic durable commodities at their post-harvest stages [6]. Nevertheless, a major shortcoming of the addition of DEs into stored grains is the need for dose rates much higher than those of conventional insecticides, i.e., doses that often may exceed 1000 ppm (1 kg DE per ton^−1^ of grain) [7]. In this context, several modified formulations that can be effective at lower concentrations have been tested with often contradictory results [8,9]. Another way to increase DE efficacy while keeping their dose rates low is to apply them simultaneously with other agents, such as neurotoxic insecticides [9,10]. For instance, Korunic et al. [10] applied a formulation of a DE with small amounts of deltamethrin and reported a high residual efficacy against the rice weevil, *Sitophilus oryzae* (L.) (Coleoptera: Curculionidae), the lesser grain borer, *Rhyzopertha dominica* (F.) (Coleoptera: Bostrychidae), and the red flour beetle, *Tribolium castaneum* (Herbst) (Coleoptera: Tenebrionidae), even 12 months after the treatment. Nevertheless, the addition of insecticides diminishes the potential of the use of DEs in organic agriculture and can complicate the registration process of such a formulation.

One additional way to decrease DE dose rates without affecting their efficacy against stored-product insects is the modification of certain key physicochemical properties, such as their particle size [2,11,12,13]. In an earlier study, Vayias et al. [13] found that the efficacy of DEs obtained from natural deposits of different areas of South Eastern Europe against *S. oryzae*, *R. dominica*, and the rusty grain beetle, *Cryptolestes ferrugineus* (Stephens) (Coleoptera: Laemophloeidae), was negatively correlated with their particle size. More recently, Baliota and Athanassiou [2] tested specific modifications of DEs obtained from Greece and found that some of these modifications drastically increased their insecticidal efficacy. Specifically, in that study, the authors reported that raw DE materials that were not initially effective could be modified through sieving, to obtain a homogenous particle size, and drying, increasing in that way their efficacy for the control of several stored-product insect species [2]. Specifically, DE formulations with <20 μm particle size were more effective than large ones when admixed with grains [2]. Similar results regarding the efficacy of these formulations have also been reported in the case of surface treatments [14].

One important characteristic of DEs is their residual effect, which, in contrast with most traditional grain protectants, is highly desirable [3,4,15]. Athanassiou et al. [15] tested three commercial DE formulations on wheat and barley and found that these formulations could be effective for the control of *S. oryzae* for at least 270 days. This is partially due to the fact that DE particles do not interact much with the external kernel part, retaining their insecticidal effect for a long time without significant dissipation [6]. Nevertheless, there might be some negative interactive effects with the kernels of certain grains [15,16,17]. Indicatively, it has been shown that DEs are more effective on wheat than on maize, partially due to the inability of maize kernels to retain the DE particles [15,17]. Thus, a DE concentration that is effective for the control of insects in some grains, may not be effective on other grains, and higher doses may be required in the case of the latter. Still, most data available for the residual effect of DEs are based on commercial formulations, while there are not many data available in the case of raw DEs.

Considering the above and taking into account the increased efficacy of the deposits that have been tested in the study of Baliota and Athanassiou [2], the aim of the current study was to test the residual effect of two of these DEs on wheat and maize. In this effort, we used two primary colonizers, *S. oryzae* and *R. dominica*. This study attempts to provide more evidence for the successful utilization of these natural insecticides for long-term protection of stored products from insect infestations, as an alternative to residual insecticides and fumigants that are commonly used in stored-grain protection systems or as part of integrated pest management (IPM) strategies.

## 2. Materials and Methods

### 2.1. DE Deposit Used

This study assessed the effectiveness of the two most effective DE formulations identified in a prior research work conducted by Baliota and Athanassiou [2]. Briefly, an initial natural DE deposit was obtained from a single mine located in the Prefecture of Thessaly in Central Greece, in the area of Elassona. A basic process of milling and drying and then a process of separating the diatoms from other elements was followed, to create two new, enriched in diatoms and with variations in their particle size DE powders, i.e., DE5 and DE6, formulations. DE5 contains 70% of semifractured diatoms and 80% of particles smaller than 45 μm, while DE6 has 68% of totally fractured diatoms and 99% of particles smaller than 45 μm. More details regarding the characteristics of DE5 and DE6 can be found in the work of Baliota and Athanassiou [2]. The DEs were stored in the laboratory at ambient conditions, until the beginning of the experiments.

### 2.2. Insect Species and Commodity Tested

Mixed-sex, less than a month old, adults of *S. oryzae* and *R. dominica* were used in the bioassays. All insects were taken from standard laboratory cultures maintained at the Laboratory of Entomology and Agricultural Zoology of the University of Thessaly for more than 15 years. Both species are reared on whole soft wheat kernels at 26 ± 1 °C and 56 ± 5% relative humidity (RH).

### 2.3. Bioassays

The bioassays were carried out in the controlled conditions chambers of the Laboratory of Entomology and Agricultural Zoology. Three different lots of 4 kg of (soft) wheat and maize were transferred in glass jars of 1 L capacity (Bormioli Luigi S.p.A., Viale Europa, 72A-43122 Parma, Italy) and were dusted with either 500 or 1000 mg kg^−1^ (ppm) of each DE, with different sets of jars for each dose and commodity. The jars were sealed with a lid and shaken manually for approximately 15 min to equally distribute the DE particles in the kernels. A series of untreated 4 kg lots of wheat and maize served as controls.

Right after the shaking, three samples of 20 g of the treated grain were transferred from each jar to three cylindrical plastic vials (3 cm in diameter, 8 cm high, Rotilabo Sample tins Snap-on lid, Carl Roth, Schoemperlenstraße 3-5, D-76185 Karlsruhe, Germany), with different series of vials per commodity. Then, twenty adults (<month old) of each species were introduced into each vial with different series of vials per insect species. Since the whole process was repeated three times, creating new jars of treated commodity each time, there were three replicates (jars) with three sub-replicates (vials), i.e., 9 vials for each combination (species–commodity–DE type–DE dose). The entire process was repeated on a monthly basis for 6 consecutive months. The jars containing the initial quantities of treated kernels were shaken monthly, for 10 min, after which samples of grains were transferred to new vials together with new adults. Insect mortality was evaluated after 7, 14, and 21 days of exposure to each treated commodity, where dead adults were removed from the vials. The jars along with the vials with the insects were always kept at controlled conditions of 26 ± 1 °C and 56 ± 5% (RH), in continuous darkness.

### 2.4. Statistical Analysis

Before the analysis, all data were assessed for assumptions of normality using the Shapiro–Wilk Test and for homoscedasticity using Levene’s Test. The assumptions were met for parametric analysis of all data. Then, the data were subjected to MANOVA [18] with time interval as the repeated factor, insect mortality as the response variable, and DE type, dose, commodity, and DE application period as the main effects. When significant differences were detected, post hoc comparisons using the Tukey–Kramer (HSD) test followed to compare means at α = 0.05. All analyses were performed using the JMP^®^ Software (version 7.0) (SAS Institute Inc., Cary, NC, USA).

## 3. Results

For all tested species, all main effects and associated interactions were significant, with some exceptions (Table 1). Generally, although *S. oryzae* was affected by all main effects (commodity, DEs, and dose), *R. dominica* responded similarly in the tested DEs. Nevertheless, the interaction of the DEs with other main effects was found to be significant for *R. dominica* (Table 1).

**Figure 1:**
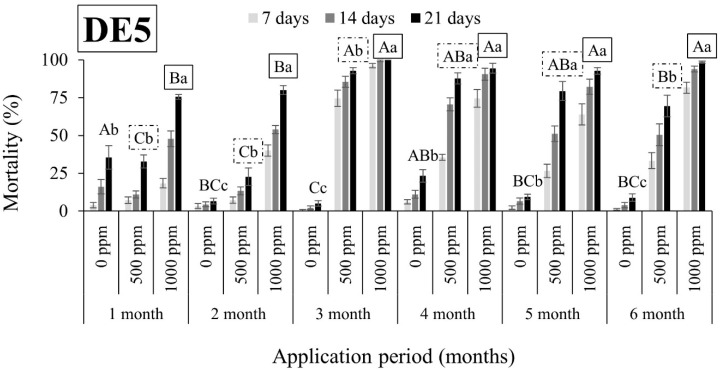
Mean mortality (% ± SE) of *Sitophilus oryzae* adults after 7, 14, and 21 days of exposure to untreated wheat and wheat treated with two different doses of DE5 (500 and 1000 ppm) for 6 consecutive months after application. Means for mortality after 21 days of exposure followed by the same lowercase letter are not significantly different among the three doses within each evaluation interval (1–6 months) according to Tukey-HSD test at α < 0.05. Means of mortality after 21 days of exposure followed by the same uppercase letter and box (no box, dash or solid box) are not significantly different among evaluation intervals (1–6 months) within each dose (0, 500, and 1000 ppm) according to Tukey-HSD test at α < 0.05.

**Figure 2:**
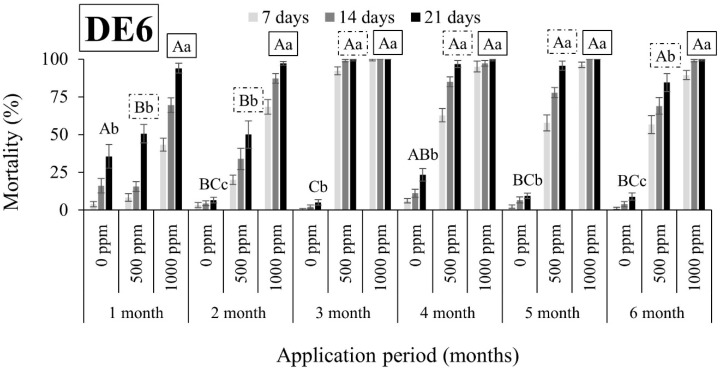
Mean mortality (% ± SE) of *Sitophilus oryzae* adults after 7, 14, and 21 days of exposure to untreated wheat and wheat treated with two different doses of DE6 (500 and 1000 ppm) for 6 consecutive months after application. Means for mortality after 21 days of exposure followed by the same lowercase letter are not significantly different among the three doses within each evaluation interval (1–6 months) according to Tukey-HSD test at α < 0.05. Means of mortality after 21 days of exposure followed by the same uppercase letter and box are not significantly different among evaluation intervals (1–6 months) within each dose (0, 500, and 1000 ppm) according to Tukey-HSD test at α < 0.05.

**Figure 3:**
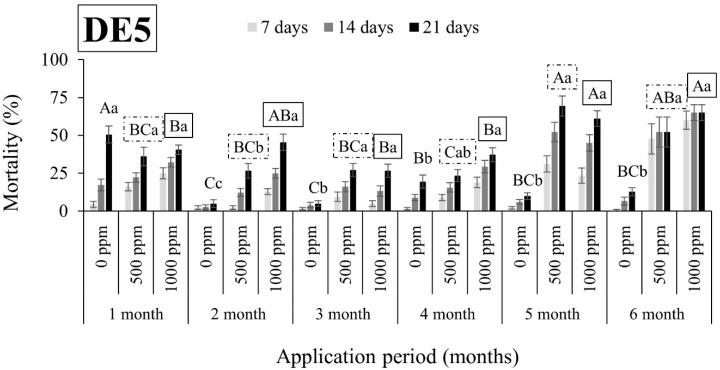
Mean mortality (% ± SE) of *Sitophilus oryzae* adults after 7, 14, and 21 days of exposure to untreated maize and maize treated with two different doses of DE5 (500 and 1000 ppm) for 6 consecutive months after application. Means for mortality after 21 days of exposure followed by the same lowercase letter are not significantly different among the three doses within each evaluation interval (1–6 months) according to Tukey-HSD test at α < 0.05. Means of mortality after 21 days of exposure followed by the same uppercase letter and box are not significantly different among evaluation intervals (1–6 months) within each dose (0, 500, and 1000 ppm) according to Tukey-HSD test at α < 0.05.

**Figure 4:**
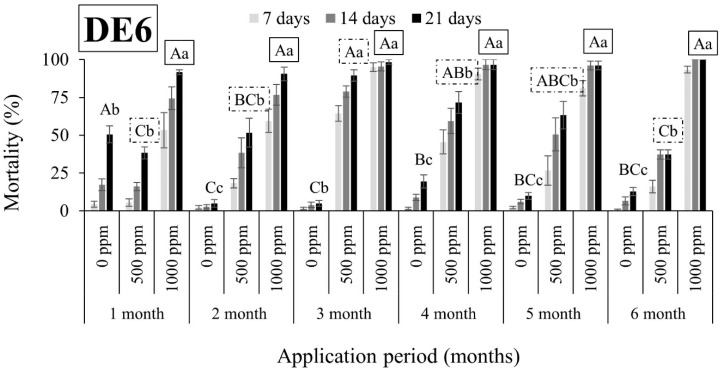
Mean mortality (% ± SE) of *Sitophilus oryzae* adults after 7, 14, and 21 days of exposure to untreated maize and maize treated with two different doses of DE6 (500 and 1000 ppm) for 6 consecutive months after application. Means for mortality after 21 days of exposure followed by the same lowercase letter are not significantly different among the three doses within each evaluation interval (1–6 months) according to Tukey-HSD test at α < 0.05. Means of mortality after 21 days of exposure followed by the same uppercase letter and box are not significantly different among evaluation intervals (1–6 months) within each dose (0, 500, and 1000 ppm) according to Tukey-HSD test at α < 0.05.

**Figure 5:**
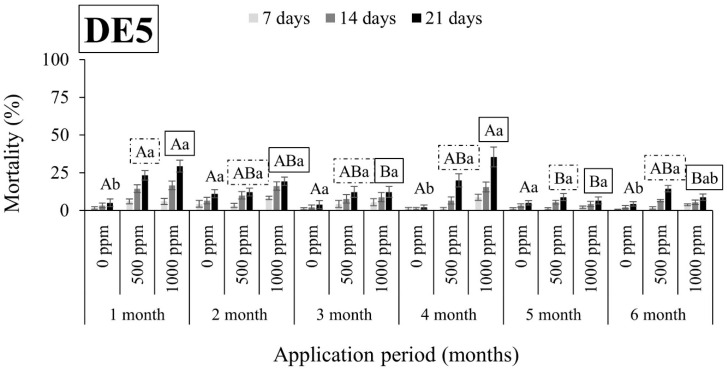
Mean mortality (% ± SE) of *Rhyzopertha dominica* adults after 7, 14, and 21 days of exposure to untreated wheat and wheat treated with two different doses of DE5 (500 and 1000 ppm) for 6 consecutive months after application. Means for mortality after 21 days of exposure followed by the same lowercase letter are not significantly different among the three doses within each evaluation interval (1–6 months) according to Tukey-HSD test at α < 0.05. Means of mortality after 21 days of exposure followed by the same uppercase letter and box are not significantly different among evaluation intervals (1–6 months) within each dose (0, 500, and 1000 ppm) according to Tukey-HSD test at α < 0.05.

**Figure 6:**
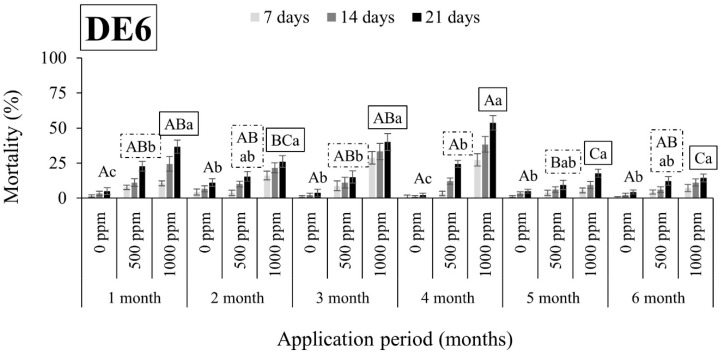
Mean mortality (% ± SE) of *Rhyzopertha dominica* adults after 7, 14, and 21 days of exposure to untreated wheat and wheat treated with two different doses of DE6 (500 and 1000 ppm) for 6 consecutive months after application. Means for mortality after 21 days of exposure followed by the same lowercase letter are not significantly different among the three doses within each evaluation interval (1–6 months) according to Tukey-HSD test at α < 0.05. Means of mortality after 21 days of exposure followed by the same uppercase letter and box are not significantly different among evaluation intervals (1–6 months) within each dose (0, 500, and 1000 ppm) according to Tukey-HSD test at α < 0.05.

**Figure 7:**
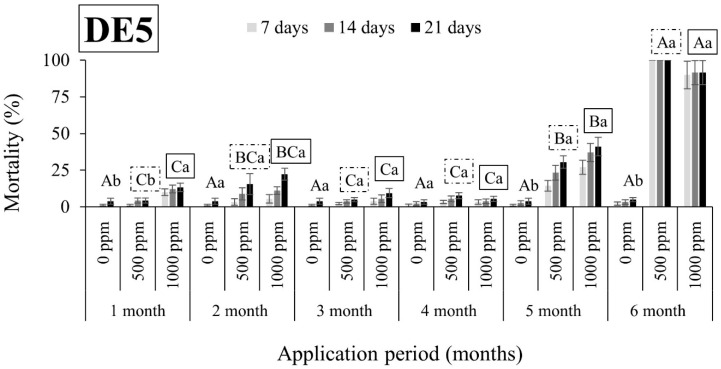
Mean mortality (% ± SE) of *Rhyzopertha dominica* adults after 7, 14, and 21 days of exposure to untreated maize and maize treated with two different doses of DE5 (500 and 1000 ppm) for 6 consecutive months after application. Means for mortality after 21 days of exposure followed by the same lowercase letter are not significantly different among the three doses within each evaluation interval (1–6 months) according to Tukey-HSD test at α <0.05. Means of mortality after 21 days of exposure followed by the same uppercase letter and box are not significantly different among evaluation intervals (1–6 months) within each dose (0, 500, and 1000 ppm) according to Tukey-HSD test at α <0.05.

**Figure 8:**
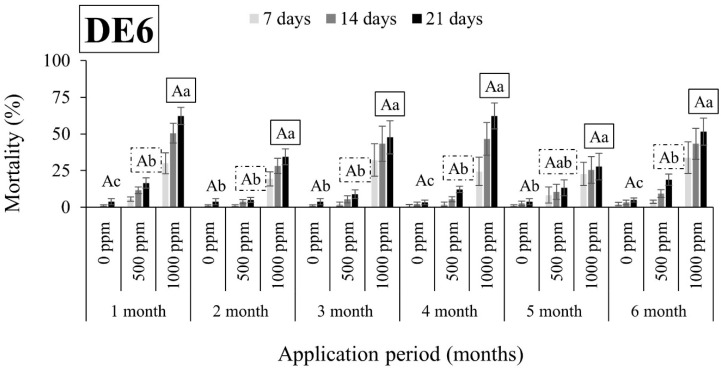
Mean mortality (% ± SE) of *Rhyzopertha dominica* adults after 7, 14, and 21 days of exposure to untreated maize and maize treated with two different doses of DE6 (500 and 1000 ppm) for 6 consecutive months after application. Means for mortality after 21 days of exposure followed by the same lowercase letter are not significantly different among the three doses within each evaluation interval (1–6 months) according to Tukey-HSD test at α < 0.05. Means of mortality after 21 days of exposure followed by the same uppercase letter and box are not significantly different among evaluation intervals (1–6 months) within each dose (0, 500, and 1000 ppm) according to Tukey-HSD test at α < 0.05.

As shown in Figure 1, Figure 2, Figure 3, Figure 4, Figure 5, Figure 6, Figure 7 and Figure 8, both DEs maintained their insecticidal effectiveness consistently without any decline over time. Six months after the DE application on wheat, mortality rates did not change for *S. oryzae* (Figure 1, Figure 2, Figure 3 and Figure 4). In fact, there was a notable increase in the mortality of the species when insects were exposed to wheat that had been treated with DE5 and DE6 before three to six months, as compared to wheat treated before one to two months, regardless of the dose tested (Figure 1 and Figure 2). The specific cause for this pattern is uncertain. On the other hand, the effects of the DE doses on *S. oryzae* mortality were also significant, but this factor was found to be DE type-dependent. More specifically, for DE5, the higher dose (1000 ppm) was significantly in most cases more effective in controlling the species than the lower dose (500 ppm) (Figure 1). However, for DE6, both doses of 500 and 1000 ppm caused high mortality (close to 100%) to *S. oryzae*, especially 3 months after application (Figure 2). The lower efficiency of DE5 against *S. oryzae* was also observed when insects were exposed to treated maize (Figure 3). In all cases, the mortality rates did not exceed 70% (Figure 3), while DE6 caused 90 to 100% mortality of *S. oryzae*, but only when applied at the higher dose (Figure 4).

Overall, *R. dominica* was found to be tolerant to DEs, since the mortality rates were in all cases lower than 63% (Figure 5, Figure 6, Figure 7 and Figure 8). An exception to this trend was observed in the case of DE5 in maize 6 months after application, with mortality rates exceeding 90% at both doses (Figure 7). However, the authors remain skeptical about this phenomenon, which may have contributed to other factors.

## 4. Discussion

Our results illustrate that the DEs tested here were more effective against *S. oryzae* than against *R. dominica*. Several studies show that *R. dominica* is less agile than *S. oryzae*, and thus, receives fewer DE particles when it is in contact with the treated grain [3]. Fields and Korunic [19] and Subramanyam and Roesli [20] noted that this reduced susceptibility of *R. dominica* was observed in more than one DE formulation. However, there are additional reports that show that this species is susceptible to DEs [2,3]. Several factors may account for this inconsistency, such as the varying insecticidal efficacy of DE formulations or the physiological, morphological, and biological characteristics of the targeted individuals [2,12]. The current results demonstrate that the DEs tested here were not effective for the control of *R. dominica* adults, even at the highest dose tested. On the other hand, *S. oryzae* adults are considered susceptible to DEs in contrast to the larvae of these species that are not affected by DE particles, as they develop within the kernels [21].

Interestingly, there were considerable variations in insect mortality among the different evaluation intervals during the grain storage period. While we are unaware of the causes of these variations, we assume that this could be attributed to the fact that we tested different batches of adults every time, of mixed sex and, especially, age, which means that insect stress due to exposure to the DE particles might have been different among these batches [22]. This may partially explain why in some bioassays that were carried out late in the storage period, adult mortality was higher in comparison to the bioassay that was conducted in Month 1. Korunic et al. [10] tested the combined use of DE with deltamethrin and found that there were similar variations among bioassays in the mortality of *S. oryzae*, *R. dominica*, and *T. castaneum* adults. One additional explanation for the variations observed here could be the uneven distribution of DE particles in the treated grain, which resulted in different concentrations among bioassays. The effect of uneven distribution of DE particles has been discussed by Subramanyam and Roesli [20]. Uneven distribution of different grain protectants and their effects on insect control has been tested for a wide range of active ingredients that have been evaluated as grain protectants [23,24,25].

According to the observed moralities, DE6, especially when applied at 1000 ppm, might be equally effective in both wheat and maize, at least in the case of *S. oryzae*. This is an important finding, considering earlier studies that show that DEs are not effective on maize [15,17]. This was observed throughout the entire experimental period, which may suggest that there were not considerable interactions with the external kernel parts capable of reducing DE insecticidal efficacy. Similar data have been reported by other studies [3,4], where the insecticidal effect of the DEs tested was mostly affected by the dose rate, rather than the storage period or the grain type. This is also true in the case of DE5, which was more effective at 1000 than at 500 ppm, but only in the case of *S. oryzae* in wheat.

The increased effectiveness of DE6 in comparison to DE5 could have potentially contributed to the differences in the diatom granulometry between these two DEs; although both formulations originated from the same initial deposit, DE6 had 99% of diatoms smaller than 45 μm in comparison with the corresponding 80% of DE5. The study by Baliota et al. [2] confirmed the impact of granulometry and diatom percentage on the insecticidal effectiveness of the same DE powders, as well as the possibility that altering these parameters could affect the powder’s efficacy. In addition, Chiu [11] and McLaughlin [26] were the first who suggested that DE particle size may play an important role in their insecticidal value, while additional authors added more data that confirmed this observation [2,11,12,13]. Currently, commercial DE formulations are made by a simple procedure of reducing moisture and adjusting the particle size, resulting in formulations with substantial physicochemical diversity, affecting insecticidal and commodity qualities. To standardize the manufacturing of DE formulations with the best insecticidal properties, more specialized processing procedures must be established. Furthermore, the persistence and efficacy of DEs must be further examined under different application scenarios.

## 5. Conclusions

The current investigation assessed the insecticidal activity of two DE formulations derived from the same deposit but with different percentages and granulometries of diatoms against *R. dominica* and *S. oryzae* in wheat and maize. The results reported here suggest that natural diatomaceous earth deposits can constitute efficient insecticides, provided that some minor modifications take place, such as sieving and drying of the raw deposit. Overall, the DE efficiency was found to be dependent upon the particle size of the diatoms and the insect species involved. The DEs tested here can be effective for the control of *S. oryzae* but not of *R. dominica*. If grains are to be stored for a long period, 1000 ppm should be the suggested dose, as opposed to 500 ppm, which may be related to increased progeny. Finally, in contrast to other studies, we found that DE formulations can be effective on maize. Overall, this work, along with the data that have been already reported from previously published works with the same DE types, shows that different amorphous deposits can be drastically “modified” with simple techniques in order to increase their insecticidal efficacy for the control of stored-product insects.

## Figures and Tables

**Table 1:** Repeated Measures MANOVA for main effects and interactions (for each species total df = 576).

Effect (Source)		*S. oryzae*			*R. dominica*		
		df	F	*p*	df	F	*p*
All Between Interactions	Intercept	1	9763.4	<0.01	1	1093.3	<0.01
	Commodity	1	244.5	<0.01	1	71.2	<0.01
	DE	1	373.0	<0.01	1	2.7	0.09
	Commodity DE	1	54.9	<0.01	1	13.1	<0.01
	Dose	2	1861.5	<0.01	2	231.1	<0.01
	Commodity × Dose	2	67.9	<0.01	2	27.4	<0.01
	DE × Dose	2	126.1	<0.01	2	42.4	<0.01
	Commodity × DE × Dose	2	58.6	<0.01	2	12.2	<0.01
	Application month	5	68.3	<0.01	5	25.3	<0.01
	Commodity × Application month	5	25.7	<0.01	5	53.9	<0.01
	DE × Application month	5	12.4	<0.01	5	38.4	<0.01
	Commodity × DE × Application month	5	18.4	<0.01	5	28.2	<0.01
	Dose × Application month	10	47.9	<0.01	10	9.5	<0.01
	Commodity × Dose × Application month	10	6.7	<0.01	10	13.2	<0.01
	DE × Dose × Application month	10	4.7	<0.01	10	11.3	<0.01
	Commodity × DE × Dose × Application month	10	6.3	<0.01	10	7.9	<0.01
All Within Interactions	Time	2	884.9	<0.01	2	372.5	<0.01
	Time × Commodity	2	2.8	0.06	2	9.8	<0.01
	Time × DE	2	3.8	0.02	2	9.1	<0.01
	Time × Commodity × DE	2	7.0	<0.01	2	6.2	<0.01
	Time × Dose	4	40.1	<0.01	4	40.1	<0.01
	Time × Commodity × Dose	4	5.8	<0.01	4	2.8	0.02
	Time × DE × Dose	4	6.1	<0.01	4	8.8	<0.01
	Time × Commodity × DE × Dose	4	2.3	0.05	4	3.0	0.01
	Time × Application month	10	35.7	<0.01	10	7.7	<0.01
	Time × Commodity × Application month	10	3.7	<0.01	10	2.9	<0.01
	Time × DE × Application month	10	4.5	<0.01	10	3.4	<0.01
	Time × Commodity × DE × Application month	10	0.6	0.84	10	4.5	<0.01
	Time × Dose × Application month	20	11.9	<0.01	20	3.8	<0.01
	Time × Commodity × Dose × Application month	20	5.9	<0.01	20	2.0	<0.01
	Time × DE × Dose × Application month	20	1.7	0.03	20	1.5	0.06
	Time × Commodity × DE × Dose × Application month	20	1.6	0.04	20	2.1	<0.01

## Data Availability

Dataset available on request from the authors.

## References

[1] Zeni V., Baliota G.V., Benelli G., Canale A., Athanassiou C.G. (2021). Diatomaceous earth for arthropod pest control: Back to the future. Molecules.

[2] Baliota G.V., Athanassiou C.G. (2020). Evaluation of a Greek diatomaceous earth for stored product insect control and techniques that maximize its insecticidal efficacy. Appl. Sci..

[3] Rigopoulou M., Baliota G.V., Athanassiou C.G. (2023). Persistence and efficacy of diatomaceous earth against stored product insects in semi-field trials. Crop. Prot..

[4] Stathers T.E., Mvumi B.M., Golob P. (2002). Field assessment of the efficacy and persistence of diatomaceous earths in protecting stored grain on small-scale farms in Zimbabwe. Crop. Prot..

[5] Stathers T.E., Riwa W., Mvumi B.M., Mosha R., Kitandu L., Mngara K., Kaoneka B., Morris M. (2008). Can diatomaceous earths have potential as grain protectants for small-holder farmers in sub-Saharan Africa? The case of Tanzania. Crop. Prot..

[6] Korunic Z. (1998). Diatomaceous earths, a group of natural insecticides. J. Stored Prod. Res..

[7] Golob P. (1997). Current status and future perspectives for inert dusts for control of stored product insects. J. Stored Prod. Res..

[8] Delgarm N., Ziaee M., McLaughlin A. (2020). Enhanced-Efficacy Iranian Diatomaceous Earth for Controlling Two Stored-Product Insect Pests. J. Econ. Entomol..

[9] Paponja I., Rozman V., Liška A. (2020). Natural formulation based on diatomaceous earth and botanicals against stored product insects. Insects.

[10] Korunic Z., Kalinovic I., Liska A., Hamel D., Carvalho M.O., Fields P.G., Adler C.S., Arthur F.H., Athanassiou C.G., Campbell J.F., Fleurat-Lessard F., Flinn P.W., Hodges R.J., Isikber A.A. (2010). Long Term Effectiveness of the Mixture of Diatomaceous Earth and Deltamethrin on Wheat. Proceedings of the Tenth International Working Conference on Stored Product Protection.

[11] Chiu S.F. (1939). Toxicity study of so-called “inert” materials with the bean weevil *Acathoscelides obtectus* (Say). J. Econ. Entomol..

[12] Korunic Z. (1997). Rapid assessment of the insecticidal value of diatomaceous earths without conducting bioassays. J. Stored Prod. Res..

[13] Vayias B.J., Athanassiou C.G., Korunic Z., Rozman V. (2009). Evaluation of natural diatomaceous earth deposits from south-eastern Europe for stored-grain protection: The effect of particle size. Pest Manag. Sci..

[14] Baliota G.V., Athanassiou C.G. (2023). Evaluation of inert dusts on surface applications and factors that maximize their insecticidal efficacy. Appl. Sci..

[15] Athanassiou C.G., Kavallieratos N.G. (2005). Insecticidal effect, and adherence of PyriSec^®^ in different grain commodities. Crop. Prot..

[16] Korunic Z. (2016). Overview of undesirable effects of using diatomaceous earths for direct mixing with grains. Pestic. Fitomed..

[17] Kabir B.G.J., Lawan M., Jidda M.B. (2013). Bioactivity of raw diatomaceous earth against *Rhyzopertha dominica* Fab. (Coleoptera: Bostrichidae): Effects of grain type, dose rate and exposure period. IOSR J. Agric. Vet. Sci..

[18] SAS Institute Inc. (2007). Using JMP® Software, Version 7.0.

[19] Fields P., Korunic Z. (2000). The effect of grain moisture content and temperature on the efficacy of diatomaceous earths from different geographical locations against stored product beetles. J. Stored Prod. Res..

[20] Subramanyam B., Roesli R., Subramanyam B., Hagstrum D.W. (2000). Inert Dusts. Alternatives to Pesticides in Stored-Product IPM.

[21] Arthur F.H., Throne J.E. (2003). Efficacy of diatomaceous earth to control internal infestations of rice weevil and maize weevil (Coleoptera: Curculionidae). J. Econ. Entomol..

[22] Baldassari N., Prioli C., Martini A., Trotta V., Baronio P. (2008). Insecticidal efficacy of a diatomaceous earth formulation against a mixed age population of adults of *Rhyzopertha dominica* and *Tribolium castaneum* as function of different temperature and exposure time. Bull. Insectology.

[23] Daglish G.J., Nayak M.K. (2010). Uneven application can influence the efficacy of S-methoprene against *Rhyzopertha dominica* on wheat. J. Stored Prod. Res..

[24] Wakil W., Riasat T., Lord J.C. (2013). Effects of combined thiamethoxam and diatomaceous earth on mortality and progeny production of four Pakistani populations of *Rhyzopertha dominica* (Coleoptera: Bostrichidae) on wheat, rice and maize. J. Stored Prod. Res..

[25] Awais M., Hasan M., Sagheer M., Asif M.U., Ali Q., Zaman S. (2019). Efficacy of diatomaceous earth and insect growth regulators against *Tribolium castaneum* (Herbst) (Coleoptera: Tenebrionidae). Sci. Lett..

[26] McLaughlin A. Laboratory trials on desiccant dust insecticides. Proceedings of the 6th Working Conference for Stored Products Protection.

